# Could Life Have Started on Mars? Planetary Conditions That Assemble and Destroy Protocells

**DOI:** 10.3390/life14030415

**Published:** 2024-03-20

**Authors:** Francesca C. A. Cary, David W. Deamer, Bruce F. Damer, Sarah A. Fagents, Kathleen C. Ruttenberg, Stuart P. Donachie

**Affiliations:** 1 Hawai’i Institute of Geophysics and Planetology, University of Hawai‘i at Mānoa, 1680 East-West Road, Honolulu, HI 96822, USA; cary.francesc@gmail.com (F.C.A.C.); fagents@hawaii.edu (S.A.F.); 2 Department of Biomolecular Engineering, Baskin School of Engineering, University of California, 1156 High Street, Santa Cruz, CA 95064, USA; deamer@soe.ucsc.edu; 3 School of Ocean and Earth Science and Technology, University of Hawai‘i at Mānoa, 1000 Pope Road, Honolulu, HI 96822, USA; kcr@hawaii.edu; 4 School of Life Sciences, University of Hawai‘i at Mānoa, 1800 East-West Road, Honolulu, HI 96822, USA

**Keywords:** selection, protocells, Mars, prebiotic chemistry, cations, origin of life, hot springs

## Abstract

Early Mars was likely habitable, but could life actually have started there? While cellular life emerged from prebiotic chemistry through a pre-Darwinian selection process relevant to both Earth and Mars, each planet posed unique selection ‘hurdles’ to this process. We focus on drivers of selection in prebiotic chemistry generic to Earth-like worlds and specific to Mars, such as an iron-rich surface. Iron, calcium, and magnesium cations are abundant in hydrothermal settings on Earth and Mars, a promising environment for an origin of life. We investigated the impact of cations on the stability and disruption of different primitive cell membranes under different pH conditions. The relative destabilizing effect of cations on membranes observed in this study is Ca^2+^ > Fe^2+^ > Mg^2+^. Cation concentrations in Earth systems today are too low to disrupt primitive membranes, but on Mars concentrations could have been elevated enough to disrupt membranes during surface dehydration. Membranes and RNA interact during dehydration–rehydration cycles to mutually stabilize each other in cation-rich solutions, and optimal membrane composition can be ‘selected’ by environmental factors such as pH and cation concentrations. We introduce an approach that considers how life may have evolved differently under the Martian planetary conditions and selective pressures.

## 1. Introduction

When investigating prospects for life in the solar system and on exoplanets, it is frequently assumed that life could begin on any planet or moon that is or was habitable. Habitability is centrally based upon the presence of liquid water on a planetary body at some time in its history [1]. However, the origin of life is a multi-stage process that requires a sufficiently dynamic and chemically complex environment to drive the selection and evolution of chemical compounds into life. This complexity is not necessarily a property of all habitable worlds. A set of dynamic and complex environmental factors that can facilitate an origin of life has been collectively described as ‘urability’ in one recent proposal [1], and has been alluded to in previous work [2,3,4,5]. A second proposal advocates for a broader definition of habitability, ‘genesity’, spotlighting combinatorial diversity generation, informational driving forces, and energetic driving forces as factors important to the origin of life and habitability [6]. Both proposals describe the ability of planetary environments to facilitate origins of life, and highlight the need to weave a framework for evolution into our search for ‘habitable’ worlds. Doing so is essential, as life elsewhere may not share the same chemical composition as that which has evolved on Earth, and may originate through pathways different to those that are familiar on Earth. Such novel pathways may be only characterizable through the identification and recognition of universal life processes elsewhere, such as evolution.

How life originated on Earth is not fully understood. Even less is understood about the origin of life at the phenomena-scale. Most research on the origin of life has focused on early Earth environments, such as hydrothermal deep-sea vents and hot springs locations where specific organic molecules sequestered by cellular life on Earth can be found and form structures resembling primitive cells [7,8,9,10,11]. Related approaches explain evolution towards life in chemistry by focusing on self-organization (e.g., [12,13]), prebiotic natural selection(e.g., [7,14,15,16]), molecular self-replication, auto-catalysis, and dynamic kinetic stability (e.g., [17,18,19,20,21,22]), more broadly. Additionally, advances in theoretical origin-of-life research provide frameworks that characterize fundamental life processes, expanding origin-of-life models from the origin of cells to the origin of information, selection, complexity, and evolution (e.g., [23,24,25,26]). All approaches are necessary to cohesively examine the origin of life.

**Figure 1:**
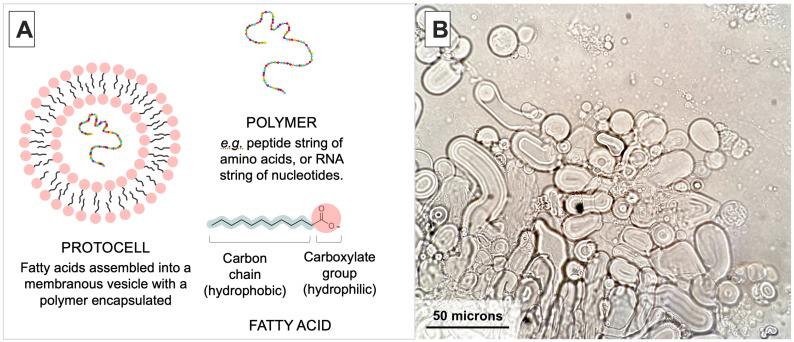
(**A**): Schematic diagram of a protocell. The structure of self-assembled fatty acids with encapsulated polymers is called a ‘protocell’. The contents and morphology of individual protocells are unique in lab and field settings [7,27]. Some protocells have characteristics that make them more physically robust than others, which forms the conceptual basis for protocells to undergo a primitive version of natural selection at a macro-molecular scale. (**B**): Micrograph of diverse fatty acid vesicle morphology in the presence of RNA. The size of membrane vesicles can vary from a few microns to 60 microns long. Vesicles can be spherical or tubular, and encapsulate various constituents from the surrounding solution, e.g., polymers and other lipids vesicles. Vesicles were made from lauric acid and glycerol monolaurate mixed with yeast RNA in a 4:1 weight/weight ratio in TEA buffer (pH 7.5). The solution was heated to ~40 °C and vortexed for 3 s. A 20 μL sample was dried on a microscope slide, then rehydrated with buffer to allow vesicles to encapsulate RNA [7,27]. After situating a cover slip on the slide, the preparation was photographed at 400× magnification. Note: an RNA stain was not used to confirm the localization of RNA inside these vesicles; see Section 2.3 for this demonstration.

For the purposes of this study, we empirically apply the hot spring hypothesis for an origin of life [7] to Mars. The hot spring scenario describes a land-based, volcanic hydrothermal field environment where cycles of evaporation and precipitation provide a mechanism for key prebiotic reactions, such as the synthesis and encapsulation of polymers inside membranous vesicles [7]. The resulting structures are referred to as ‘protocells’ (Figure 1) [7,27] and they can aggregate into proto-cellular networks that are speculated to be collectively capable of a primitive version of Darwinian Natural Selection and evolution based upon diversity and the differential survivability of protocells through environmental cycles [7,16]. Protocells are a leading model for the earliest phase of cellular life on Earth and potentially on similar planets, such as Mars. We speculate that the highly dynamic nature of hydrothermal fields is important in driving the evolution of protocells into primitive forms of life. The networked landscape of hydrothermal fields allows protocells to be continuously exposed to diverse chemical conditions, such as variable pH and ionic composition, due to local volcanic activity. Additionally, hot-spring pools provide a three-way interface between rock, atmosphere, and water, allowing wet–dry cycling to occur. Wet–dry cycling can ‘test’ the survivability of each unique protocell with each cycle, and ‘select’ for persistent prebiotic structures that emerge through time [7,16]. We consider selection in prebiotic chemistry to be analogous to Darwinian Natural Selection, whereby the interplay between the intrinsic characteristics of an individual and the environment in which it resides determine ‘survival’. In the case of modern biology, unique characteristics of organisms that allow them to produce more offspring than others are selected and are retained by the subsequent offspring. In the case of prebiotic chemistry, ‘survival’ equates to ‘persistence’. At this prebiotic level, unique molecules (e.g., autocatalytic sets of chemical reactions [20]) and supramolecular structures (i.e., lipid vesicles with encapsulated polymers [7] or reinforced membranes) that physically persist are selected for.

The origin of cellular life in volcanic hydrothermal fields is also a likely scenario for Mars [1]. Various Martian land-based volcanic hydrothermal sites have been identified [28,29,30]; however, sites similar to the deep-sea hydrothermal vents known to Earth have yet to be conclusively discovered in the northern hemisphere of Mars, where it is possible that an ocean formed [29]. Additionally, Mars lacked extensive plate tectonics; a geologic process that drives the formation of widespread deep-sea hydrothermal vents on Earth [31]. Even if some vents can be formed by magmatic hotspots [32], the origin of life is considered to be a transition that occurs on a planetary scale [30,33,34]. There is reason to believe that oceanic hydrothermal vents would not have been the dominant hydrothermal setting on Mars. Therefore, we adopt the scenario of volcanic hot springs as potential origin-of-life sites to investigate Martian prebiotic selection processes in this study.

Given the broad similarities between early Earth and early Mars, the two planets are proposed to have had comparable urability [1,2,6,29]. Both planets show evidence of volcanism, liquid water, substantial atmospheres, light, and chemical energy sources early in their history. Additionally, the chemical elements and organic molecules that were sequestered by life on Earth are thought to have been available on early Mars [1,2]. Taken together, these factors make it plausible that life originating on Mars had ingredients to work with that were compositionally similar to those on Earth, and such molecular ingredients would have been compatible with aqueous environments. However, it is essential to critically assess the urability (and genesity) of Mars before assuming that life was able to originate there, inhabit Martian surface lakes, and take a familiar form.

Evidence that life existed on Earth ~1 billion years post-accretion is preserved in geological samples [35], and this timeframe is frequently referred to as plausible for an origin of life on Mars. Because the surface of early Mars was similar to that of the early Earth for roughly 1–1.5 billion years post-accretion [29], it is often assumed life could have originated on Mars and become similarly preserved in the Martian rock record [36]. To some degree, extrapolating the conditions of early Earth to Mars is reasonable, and it is possible that life started on Mars in this time. However, basing this assumption on the timeline alone, rather than the urable processes occurring within that timeline, severely limits our understanding of the extent to which Mars could have facilitated an origin of life and how different the result could have been. Rather than considering whether life could have existed on Mars based on assumed similarities to Earth, this research considers whether life could have originated on Mars, by evaluating differences between specific urability factors on early Mars and Earth and experimentally assessing their impact on protocell formation and evolution.

**Figure 2:**
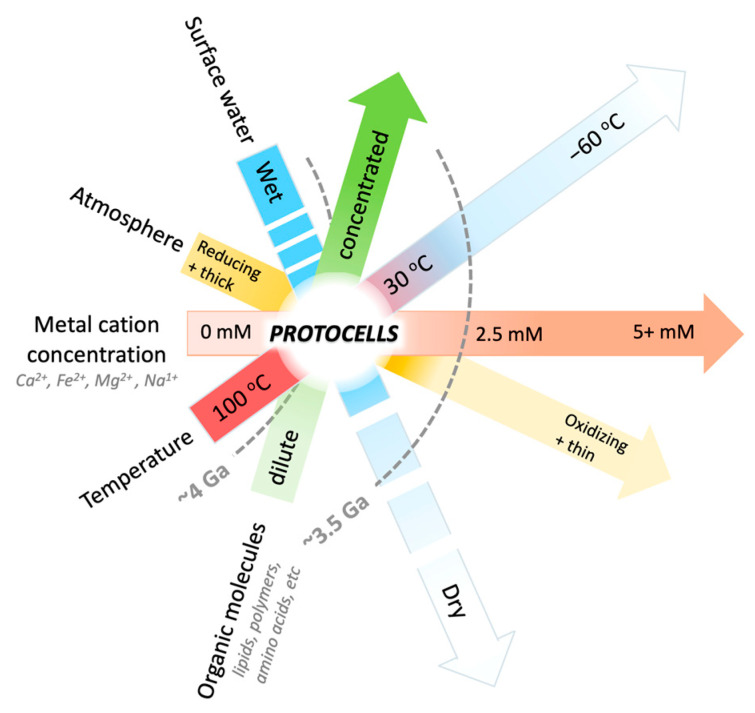
Urable Mars? Schematic representation of the atmospheric, hydrological, and geochemical conditions that would have favored protocell formation on early Mars (‘urable’ conditions). Each bar represents a range of environmental conditions. Combined space where bars overlap represent conditions under which protocells can form. White lines on the surface-water bar indicate cycles of hydrated and dehydrated conditions, including short-term (hours, days, or weeks) and long-term (years, thousands of years, or millions of years) cycling in times of episodic surface water on Mars. Mars is speculated to have been a favorable environment for protocell formation ~4 Ga [1], then shifted out of such urable conditions ~3.5 Ga (gray dashed line) when a drying event resulted in the loss of the atmosphere and surface water. From that time forward, the evolution from protocells to living cells would have been more challenging. Urability graph adapted from [1].

The surface conditions of Earth and Mars have diverged over the past four billion years, creating significant geochemical differences that are relevant to the origin of cellular life. A relatively abrupt change in Martian planetary surface conditions occurred between ~3.5 and 3 Ga (Figure 2) [37], including atmospheric escape, the loss of surface water, and a geochemical and atmospheric shift in redox states [30,38,39,40]. These changes would have made protocell formation on the surface of Mars challenging [1] (Figure 2). If protocells or early living cells had acquired the molecular machinery to adapt to these changing surface conditions, or to retreat into the subsurface, they may have survived this shift. However, the degree of change in Martian planetary conditions over time may have been an adaptive challenge too great for even well-established cellular life to endure.

One globally evident geochemical difference between Mars and Earth is the higher iron content in the Martian crust and mantle [41,42,43], with the Martian mantle being twice as enriched in iron as the mantle of Earth [17]. Observations of surface geology through remote sensing, in situ rover, and lander exploration, as well as Martian meteorite samples, support our understanding of Mars as an iron-rich planet [41,42,43]. Iron can be mobilized from surface minerals and become incorporated into hydrothermal systems, e.g., Fe^2+^ cations are leached from iron-bearing minerals such as olivine during dissolution in the presence of carbonic acid (Fe_2_SiO_4_ + 4H_2_CO_3_ → 2Fe^2+^ + 4HCO^3–^ + H_4_SiO_4_) [44]. Ferric iron (Fe^3+^) is widespread on the oxidizing conditions of Mars today, but ferrous iron (Fe^2+^) is thought to have dominated on early Mars under a reducing atmosphere [39,45]. Fatty acids contain negatively-charged hydrophilic carboxylate (COO−) groups that are sensitive to collapse in the presence of divalent cations such as Ca^2+^ and Mg^2+^ [46], and potentially Fe^2+^ as well. Dissolved cations in solution thus impact the self-assembly and stability of lipid vesicles and protocells [7] and, by extension, urability.

Iron is known to play a key role in biological functions such as metabolism and enzyme activity, and in a primitive world, Fe^2+^ preceded the role of Mg^2+^ in catalyzing RNA synthesis in anoxic environments [47,48,49]. If the sensitivity of protocells to geochemical challenges was overcome on an early Mars, we speculate that the high iron content of Mars could have been utilized by emerging proto-cellular networks in the catalysis of biological chemistry. By investigating the relationship between differential geochemistry and key organic molecules in protocell assembly, we can examine how the geochemical setting on early Mars compared to Earth in facilitating the assembly of cellular life.

In this study, we investigate the role that Fe^2+^ cations play in the formation, stability, and disruption of primitive cell membranes and protocells. Additionally, urable factors shaping the selection of protocell membranes more generally on Earth-like planets such as Mars are investigated. These factors include the presence and concentration of Ca^2+^ and Mg^2+^ ions, low pH conditions, membrane composition, and the presence of functional polymers in membrane self-assembly processes. This research initiates a critical assessment of whether life could start under the unique conditions of Mars, and contributes to a broader understating of the link between geochemistry and prebiotic chemistry.

## 2. Materials and Methods

Extensive research on protocell formation exists (reviewed in [50]), but to understand how life actually evolved (overcoming a series of selection hurdles) it is more informative to investigate the disassembly and disruption of protocells. The disruptive effects of changes in the environment ultimately shape the chemistry that emerges in a prebiotic version of natural selection. As environmental pressure destroys certain structures and not others, selection provides a powerful explanation for how some features emerge in a system, and is the focus of an experimental strategy in this research.

In the presence of cations, fatty acids tend to form large aggregates of destabilized vesicles instead of protocells. This is known as ‘flocculation’ [51]. In the presence of cations, the self-assembly of new fatty acid vesicles and the stability of extant ones is disrupted [51,52,53], preventing vesicles from progressing toward protocells. Instead, vesicles form ‘flocs’. A cation-rich environment poses a selective pressure on membrane vesicles, which may be destroyed if they lack flocculation-resistant physical properties. Flocculation triggered by iron-rich environments could have been a selective hurdle for protocell formation unique to Mars. Building upon previous and widely implemented methods for studying protocell assembly in volcanic hydrothermal settings and seawater solutions [27,52], this research quantitatively investigated the effect of Fe^2+^ on the formation and stabilization of lipid vesicles and protocells relative to Ca^2+^ and Mg^2+^.

Cationic abundances, fatty acid synthesis, pH, and wet–dry cycling are controlled by local geochemical and environmental conditions, and may also vary between Earth-like planets. Therefore, in addition to investigating the role that iron plays in vesicle stability compared with other cations, this research also explored the effect that fatty acid composition, acidic conditions, and wet–dry cycling in the presence of polymers have on protocell stability. For the purposes of investigating the chemical relationship between polymers and lipids, RNA is used as a model for the type of polymer that could have been present in the first protocells billions of years ago. Combined, this approach builds a foundation for assessing how protocell stability might differ between environments on Mars and Earth due to differences in geochemistry and hydrological cycling, and more broadly how the environment impacts the prebiotic chemistry that emerges from it.

### 2.1. Anaerobic Conditions

All sample preparation, experimentation, and analysis took place in ananaerobic chamber (Plas-Labs 855 AC, Lansing, MI, USA) purged with nitrogen gas (N_2_) in order to test iron in its ferrous state (+2). Buffers and water were vigorously bubbled with N_2_ and sealed prior to entry into the chamber. Seals were removed only once inside the chamber, so that during cycles of evacuating and flushing with N_2_, any residual oxygen gas in the flask would be removed. Solids were pre-weighed outside the chamber, but only dissolved in the chamber after anaerobic conditions were achieved. The chamber was sealed and flushed with nitrogen gas ~23 times until the oxygen content was 0.0% (measured with an O_2_ meter). A UV-1600PC UV-VIS spectrophotometer was housed in the chamber in order for sample analyses to be conducted only under anaerobic conditions. Note: despite extensive precaution in maintaining an anaerobic environment, we observed some oxidation and precipitation in Fe^2+^ samples. In order to address the concern about potential impacts Fe^3+^ precipitates may have on the fatty acid vesicle flocculation observed in our experiments, we ran a subsequent experiment using ferrous ammonium sulfate (Fe(NH_4_)_2_(SO_4_)_2_) for Fe^2+^ samples, and ferric ammonium sulfate (FeNH_4_(SO_4_)_2_) for Fe^3+^ samples, in order to control the oxidation state of iron. This experiment indicated that the oxidation state of iron had a negligible impact on the flocculation and light-absorbance readings in our study [53].

### 2.2. Flocculation of Lipid Vesicles in the Presence of Cations

**Figure 3:**
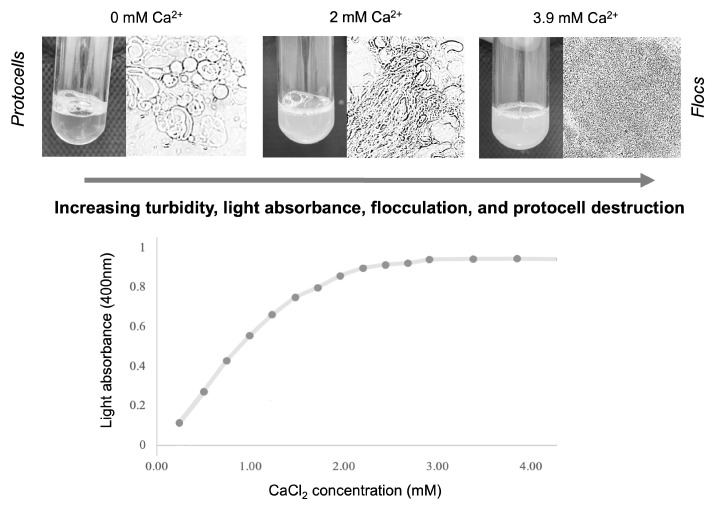
Relationship between flocculation and light absorbance. As flocculation increases in lipid solutions (LAGML, 10 mM, pH 7.5) exposed to 0–3.9 mM calcium cations, solutions become visibly more turbid and absorb more light when analyzed by spectrophotometry at 400 nm.

The flocculation of various lipid vesicle solutions in the presence of Fe^2+^ and other cations was quantified through spectrophotometry [53]. Upon the addition of cations to the solution, lipid vesicle flocculation causes the solution to become more turbid and absorb more light. Vesicle flocculation caused increases in solution turbidity visible to the naked eye, microscopy, and spectrophotometry (Figure 3). Spectrophotometry samples were analyzed at 400 nm, the wavelength at which the maximum light absorbance/minimum light transmission can be observed for lipid vesicle flocs.

Lipid vesicles solutions were prepared as described in Table 1, warmed to ~40 °C, and vortexed for 3 s. Various mixtures of lauric acid (LA), capric acid (CA), and glycerol monolaurate were used to investigate the effect of cations on vesicle stability and flocculation for different lipid combinations. Glycerol monolaurate (GML) was mixed with fatty acids, as it stabilizes the resulting lipid vesicle structures across a range of pH values and ionic compositions [54], to ensure vesicle structures persisted during experimentation. Additionally, glycerol and fatty acids form monoglycerides (e.g., glycerol monodecanoate [55]) when heated in water, representing a prebiotically plausible addition to the organic molecules available for lipid vesicle self-assembly.

Cation solutions of 100 mM iron, calcium, and magnesium were prepared in TEA (pH 7.5) or MES buffer (pH 5.5). Cation solutions were warmed to ~40 °C in a water bath, as were the vesicle solutions. Fe^2+^ samples were prepared with ferrous sulfate (FeSO_4_), Ca^2+^ with calcium chloride (CaCl_2_), and Mg^2+^ with magnesium sulfate (MgSO_4_).

Samples for which spectrophotometric data were acquired comprised 2 mL of the lipid vesicle solution pipetted into a 1 cm^2^, 3 mL, disposable plastic cuvette with a 1 cm light path. Cation solutions were mixed serially into the cuvette in 5–10 μL increments. Sample turbidity was determined spectrophotometrically at 400 nm two minutes after each serial cation addition. Triplicate samples per experimental variable were analyzed.

### 2.3. Flocculation of Lipid Vesicles with Encapsulated RNA in the Presence of Cations

**Figure 4:**
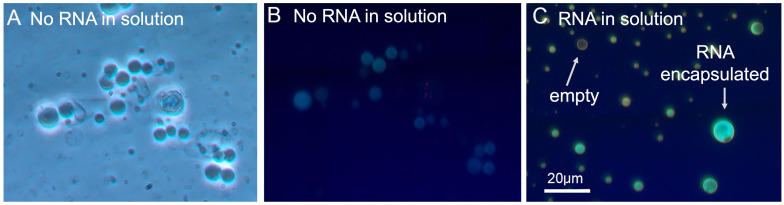
RNA captured inside membrane vesicles by a single wet–dry–wet cycle. (**A**): Phase micrograph of LAGML vesicles with acridine orange present but no RNA (control), vesicle membrane structures visible. (**B**) Fluorescent micrograph of LAGML vesicles with acridine orange present but no RNA (control), faint fluorescence of membranes visible. (**C**): Fluorescent micrograph of LAGML vesicles with encapsulated RNA, brightly fluorescent RNA visible inside vesicles. RNA Vesicles in panels A and B were prepared in 10 mM phosphate buffer, pH 7.8. LAGML (10 mg) was dissolved in chloroform in a test tube, then 2.0 mL of buffer was added. The solution was then heated to 50 °C to ensure LAGML was in a fluid phase. The vesicles were immediately dispersed by vortexing the warm solution for 15 s. To prepare vesicles with encapsulated RNA in panel C, an aliquot of the vesicle solution from panels A and B was extracted and mixed with yeast RNA (10 mg/mL in water) in a 4:1 weight ratio of lipid to RNA. Then, 20 μL of the mixture was pipetted onto a microscope slide, which was then allowed to evaporate at 85 °C before being rehydrated with 20 μL of 10 mM phosphate buffer and 1 μL acridine orange (0.19 μM in final solution). Vesicles with encapsulated RNA formed spontaneously. Credit: D. W. Deamer.

Wet–dry cycling allows lipid vesicles to encapsulate RNA [7,27] and is experimentally achieved by allowing heated solutions of lipids, RNA, and buffer to evaporate until a dry film of lipids and RNA remain. After drying, the buffer was replenished and the solution was heated. In this study, RNA-LAGML vesicle solutions were prepared by combining 0.06 g LAGML and 0.015 g Yeast ribosomal RNA (4:1 ratio of LAGML to RNA) in 5 mL TEA buffer, pH 7.5 (50 mM LAGML) (Table 1). Preliminary tests stained RNA with acridine orange to confirm LAGML-encapsulated RNA in a wet–dry–wet cycle, observed under microscopic examination (Figure 4), similarly to [7,27].

RNA-LAGML vesicle solutions were warmed to ~40 °C, vortexed for one minute, and heated to ~85 °C on a hot plate until all the liquid had evaporated. TEA buffer (25 mL) was added to the dehydrated film immediately prior to experimentation to provide a final concentration of 10 mM LAGML, and the solution was warmed again and homogenized by repeated pipetting. Cation solutions (FeSO_4_, CaCl_2_, and MgSO_4_) each at 100 mM, were prepared and added to the RNA-LAGML solution. The turbidities of the resulting samples were read at 400 nm on the spectrophotometer.

Because microscopy in an anaerobic atmosphere was not feasible, Ca^2+^ cations were chosen for microscopy experiments as they are not sensitive to the oxidizing atmosphere, unlike Fe^2+^ cations, which are unstable under oxidizing conditions. For microscopy experiments, ‘LAGML’ vesicles (100 mM) comprised of lauric acid (‘LA’) and glycerol monolaurate (‘GML’) in a 1:1 weight/weight ratio were prepared in TEA buffer (10 mM, pH 7.5) by warming to ~40 °C and vortexing for 30 s. Ca^2+^ supplied as CaCl_2_ (100 mM stock solution) was added sequentially to LAGML samples to 0 mM, 0.25 mM, 0.5 mM, 1 mM, and 2 mM CaCl_2_. Samples were observed using light or fluorescence microscopy, with a micrograph captured after each addition. Samples undergoing wet–dry cycling were allowed to evaporate on the microscope slide on a hot plate ~85 °C, and were ‘rehydrated’ with TEA buffer (one wet–dry–wet cycle). LAGML concentration was 100 mM in the final samples without RNA and 50 mM in samples with RNA due to practical difficulties associated with dissolving a high concentration of RNA.

### 2.4. Buffer Choice

Buffers for this study were used to maintain pH. While hot-spring settings on Earth display a wide range of pH conditions [52], the pH of Martian hot springs are not as well characterized. One hypothezised hot-spring setting on Mars, Columbia Hills, hosts magnesium-iron carbonate lithologies consistent with formation under a near-neutral pH [56]. Near-neutral pH conditions were chosen for the majority of our experiments. Acidic pH was included as a variable in this study to explore the pH sensitivity of cation–fatty acid binding.

To ensure metal cations were interacting with lipids as desired, it was important to choose buffers that did not interact with metals, i.e., ‘non-chelating’ buffers. Triethanolamine (TEA) buffer can chelate selected ions, including iron, but this is only observed in highly alkaline pH conditions [57]. Given that our experiments use TEA to maintain a pH of 7.5, we do not expect any buffer–metal chelating to occur in our experiments. Similarly, 2-ethanesulfonic acid (MES) buffer has negligible metal–ion binding [58], and was chosen for experimentation to maintain a pH of 5.5.

## 3. Results and Interpretations

Ca^2+^, Fe^2+^_,_ and Mg^2+^ cations are the dominant divalent cations in hydrothermal solutions [27,52]. Cation-destabilized lipid vesicles produce ‘flocculation’, a physical effect measurable using light absorbance as a function of turbidity (see Section 2). In the results that follow, a lower absorbance reading corresponds to less flocculation, and is interpreted as evidence for greater protocell survival. Conversely, a higher absorbance reading reflects greater flocculation, interpreted as unfavorable conditions for protocell formation. This relationship was discernible for each cation across experimental pH ranges, vesicle compositions, and RNA wet–dry cycling.

### 3.1. Relative Impact of Ca, Fe, and Mg on Vesicle Stability

**Figure 5:**
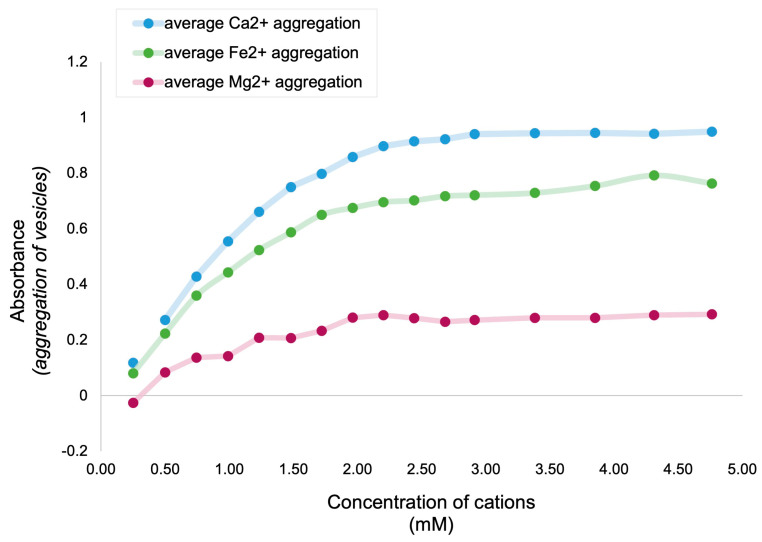
Relative absorbance (as a proxy for flocculation) of lipid vesicles triggered by Ca^2+^, Fe^2+^, and Mg^2+^ cations. Average flocculation is shown as a solid line increasing with cation concentration, with uncertainty bars representing the standard deviation for each cation in all experiments. The vesicle compositions were LAGML, LAGML pH 5.5, CAGML, LA, LACA, and RNA-LAGML (LA, lauric acid; CA, capric acid; GML, glycerol monolaurate). Data points represent the average absorbance reading across all experiments (*n* = 18) for each cation. Samples were analyzed by spectrophotometry at 400 nm.

A clear relationship is observed between the amount of fatty acid vesicle flocculation and the concentration of all cations assayed. The most extensive flocculation is triggered by the presence Ca^2+^, with Fe^2+^ and Mg^2+^ generating progressively less flocculation (Figure 5). Ca^2+^ produced the most flocculation, followed by Fe^2+^, and then Mg^2+^ (Figure 5); this is represented in the following sections as Ca^2+^ > Fe^2+^ > Mg^2+^.

#### Interpretation

The fact that the cations tested have the same ionic charge (+2) might lead one to expect a similar binding affinity to carboxylate groups (COO−) in the lipid vesicles. However, the different cations elicit different extents of fatty acid flocculation (Figure 5). Therefore, the impact of specific cations on fatty acids in solution must involve other factors. Such factors could include the ionic radius, charge density, bond length, and bond type of each cation in aqueous solutions (Table 2).

Neither Ca^2+^ or Mg^2+^ have electrons occupying their d-subshell orbitals, and both form weak electrostatic bonds with water. In contrast, Fe^2+^ has six electrons occupying its d-subshell orbital and forms strong covalent bonds with water. While each cation has a different bond strength, Ca^2+^, Fe^2+^_,_ and Mg^2+^ can all form six coordinate bonds in water. We speculate that the coordination number could vary greatly with experimental conditions such as temperature, the concentration of cations, and pH. Therefore, we instead focus on the role of the ionic radius and charge density in determining relative strength of cation coordination bonds with water and fatty acids to explain our results.

Ca^2+^ has the largest ionic radius, the longest bond distance to water, and the lowest charge density of the three cations [59,60,61,62,63,64]. We interpret this to mean that calcium can bind to more anionic sites, but the individual interactions are further away from the central metal ion, and are weaker as a result. Mg^2+^ has the smallest ionic radius, shortest bond distance to water, and has a high charge density. From this, one might expect Mg^2+^ to bind more tightly to anionic sites than Ca^2+^. While this may be true for water bound to Mg^2+^, as Mg^2+^ consistently maintains a highly hydrated state [65] and can reach ~2–5 Å [65], this may not be true for fatty acid–Mg^2+^ binding. We speculate that Ca^2+^ forms more coordinate bonds with fatty acids than Mg^2+^, evidenced by the extensive flocculation and increased light absorbance observed for Ca^2+^ (Figure 5). Given the difference in the ionic radius between Mg^2+^ and Ca^2+^, it is possible that the increased size of Ca^2+^ could accommodate more fatty acids to bind to the central metal ion than Mg^2+^, in a similar fashion to the ‘cooperative’ packing of Ethylenediaminetetraacetic acids (EDTA) [66,67] and sterate complexes [68] around large Ca^2+^ ions. The preferential binding of EDTA to Ca^2+^ over Mg^2+^ is also due to the high energetic ‘cost’ of outcompeting water molecules closely bound to Mg^2+^, and of fitting into the tight coordination environment of Mg^2+^ [66,67]. EDTA and fatty acid molecules both contain COO− groups sensitive to cations, and our results appear to be consistent with calcium having the most favorable ionic radius for COO− binding, resulting in increased flocculation (Figure 5).

Fe^2+^ generated less flocculation than Ca^2+^ but more than Mg^2+^ (Figure 5), which we speculate is attributable to its unique ionic properties. Fe^2+^ has a small ionic radius, a short bond distance to water, and a high charge density. Fe^2+^ also binds water and fatty acid COO− groups covalently in a highly specific orientation due to its d-orbital electron shell configuration. As a result, we speculate that Fe^2+^ may not bind to as many fatty acids as Ca^2+^ due to its decreased size and increased binding-orientation specificity, based on values identified in Table 2. While to our knowledge the relative binding affinity of fatty acids to Fe^2+^, Ca^2+^_,_ and Mg^2+^ has not been explicitly investigated, an appreciation of the somewhat ‘intermediate’ ionic properties of Fe^2+^ that impact its binding affinity to COO− groups offers a partial explanation for our results, and highlights an important avenue for further research.

### 3.2. Concentration of Cations in Natural Settings on Earth

**Figure 6:**
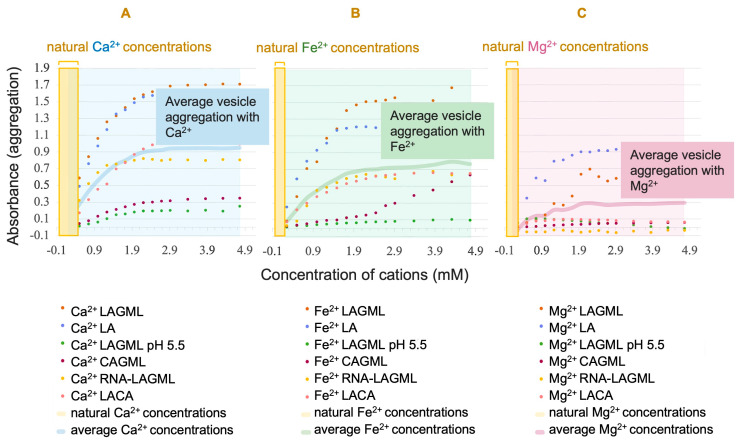
Concentration of cations in these experiments versus in natural settings. Cation concentrations for (**A**) calcium, (**B**) magnesium, and (**C**) iron, observed to produce flocculation of lipid vesicles in this study far exceed the concentrations found in natural hot-spring settings [52]. Mass flocculation of lipid vesicles occurs beyond ~1.9 mM of Ca, Fe, and Mg cations in this study, while natural settings on Earth are observed to be <0.7 mM [52,69]. Natural hot-spring data are from Midway Geyser Basin, Norris Geyser Basin, Bison Pool, and Chocolate Pots in Yellowstone California; Hell’s Gate and Whangapipiro in New Zealand; and Kamchatka in Russia. Each site had a different relative abundance of cations in solution, with the highest Fe^2+^ at Chocolate Pots in Yellowstone [52,69].

The concentrations of cations required for vesicles to reach maximum absorbance were observed to be ~1.9 mM for Ca^2+^, Fe^2+^, and Mg^2+^ (Figure 6; panels A, B, and C, respectively). The concentrations of cations in natural hot-spring water on Earth are 0.01–0.45 mM for Ca^2+^, 0.001–0.12 mM for Mg^2+^, and 0.0004–0.6123 mM for Fe^2+^ [52,69].

#### Interpretation

The concentrations of cations needed to cause a mass flocculation of vesicles, i.e., to reach maximum absorbance, exceeds the concentrations of cations found in natural hot springs, and there is little to no overlap in concentrations that produce flocculation (Figure 6). The inference is that natural hot-spring settings would have insufficient cation concentrations for lipid vesicles to flocculate, even in iron-rich hot springs such as Chocolate Pots in Yellowstone [69]. Iron-carbonate lithologies are also plausible for Martian hot springs [56], which also may have had a high relative abundance of iron.

Evaluating the relative contributions of different cations to flocculation is crucial for understanding how geochemical differences in hydrothermal systems can affect the formation of cellular life on Mars. Hot springs are affected by precipitation and evaporation cycles, which contribute to natural variability in the concentration of cations in the same pool over time as water volumes change. A potential outcome is that cation concentrations increase in pools during evaporation. Given that surface water on Mars evaporated ~3.5 Ga [30], flocculation was likely an important selective barrier for emerging protocell communities to overcome on early Earth, and more so on Mars.

### 3.3. Effect of Low pH on Vesicle Stability

**Figure 7:**
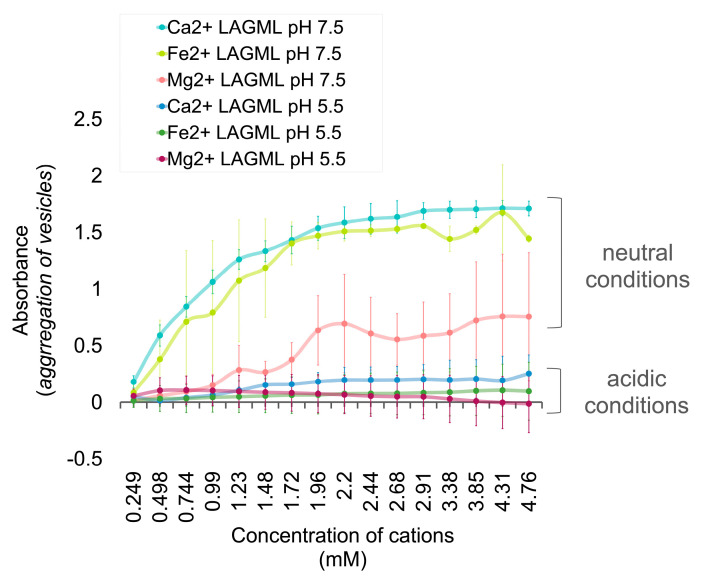
Lipid vesicle flocculation in neutral versus acidic pH. Light absorbance of lipid vesicle solutions with increasing cation concentrations, pH 7.5 (neutral conditions) and pH 5.5 (acidic conditions). High absorbance readings reflect high incidence of flocculation and unfavorable conditions for protocell formation. Samples were analyzed by spectrophotometry at 400 nm in anaerobic conditions. Uncertainty margins are calculated as the standard deviation for *n* = 3 replicates.

Under acidic conditions (pH 5.5), the absorbance readings and flocculation of LAGML vesicles (made of lauric acid and glycerol monolaurate) were lower than in neutral conditions (pH 7.5) (Figure 7). Evidence of LAGML flocculation in the presence of Ca^2+^, Fe^2+^, and Mg^2+^ is observed under both pH conditions, with the relative effect of each being consistent with the Ca^2+^ > Fe^2+^ > Mg^2+^ relationship seen previously (Figure 5).

#### Interpretation

**Figure 8:**
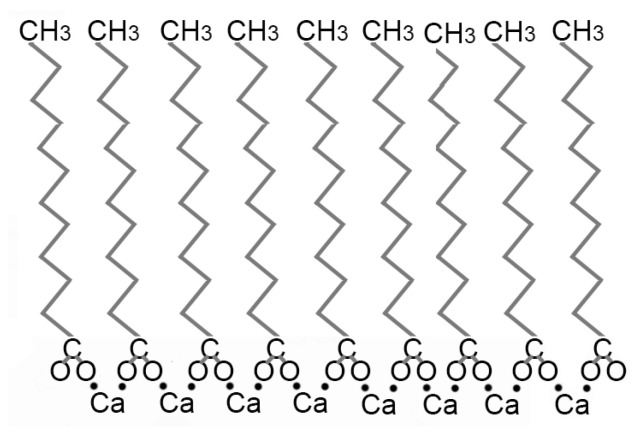
Ca^+2^ interaction with carboxylate groups on fatty acids.

pH greatly affects the morphology of lipids in solution. Protonated lauric acid forms oily droplets at low pH values, micelles at high pH, and vesicles around its pKa ~5 [45]. The interaction between carboxylate groups and metal cations is also affected by pH. Under acidic conditions, more hydronium ions are present in solution. Under a low enough pH (2–3), hydronium ions (pKa ~4) protonate carboxylate groups and neutralize their anionic charge (COO− → COOH), preventing metal cations from binding. Experimental solutions examined in this work were at pH 5.5, which would not be sufficiently acidic to protonate carboxylate groups and convert them into carboxylic acids, yet a decrease in absorbance—reflecting less flocculation—is observed under acidic conditions (Figure 7). This behavior can be understood by considering the behavior of the metal ions themselves. Metal cations bridge fatty acids and simultaneously form coordinate bonds with multiple fatty acids (Figure 8), which causes flocculation [27,46,70].

Bond formation between metal ion and ligand (such as COO−) groups is pH-sensitive [70]. We speculate that the lower absorbance reading observed for fatty acid solutions at pH 5.5 in Figure 7 is due to enhanced solubility of metal ions at low pH relative to their solubility at high pH [70]. If metal ions interact with more water molecules than fatty acid COO− groups at pH 5.5, this could explain why less flocculation and a lower light absorbance reading is observed. Note that we would expect the opposite to be true for high pH solutions, where metal ions would be less soluble and interact more with fatty acid COO− groups as observed in [27], and similarly with other small molecules such as peptides, which increasingly bind to metals at higher pH [71].

### 3.4. Effect of Fatty Acid Chain Length on Vesicle Stability

**Figure 9:**
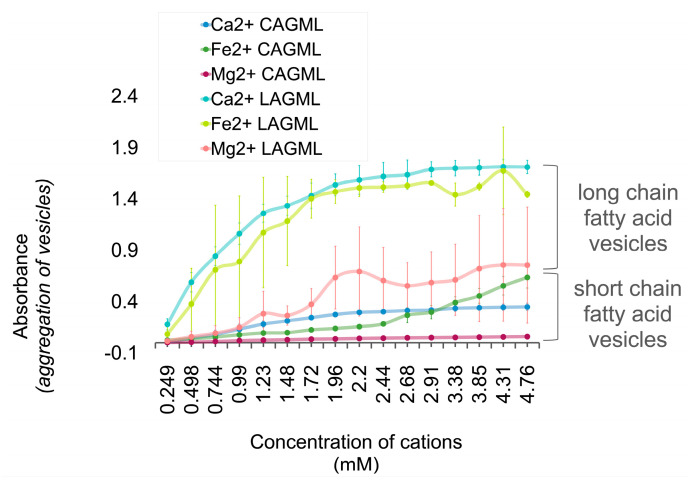
Lipid vesicle flocculation in short (CAGML)-versus long (LAGML)-chain fatty acids. Light absorbance (at 400 nm) of 10 mM LAGML and CAGML lipid vesicle solutions with increasing cation concentrations, pH 7.5. High absorbance readings reflect high incidence of flocculation and unfavorable conditions for protocell formation. Samples were analyzed by spectrophotometry under anaerobic conditions. Uncertainty margins calculated as the standard deviation for *n* = 3 replicates.

Vesicles of CAGML (capric acid and glycerol monolaurate) had lower absorbance readings, i.e., less flocculation, than vesicles of LAGML (Figure 9). Vesicles of CAGML increased in turbidity in the presence of Ca^2+^, Fe^2+^, and Mg^2+^ cations, with their relative effect on flocculation being Ca^2+^ > Fe^2+^ > Mg^2+^. However, Fe^2+^ surpassed the absorbance and flocculation produced by Ca^2+^ at concentrations of ~2.91 mM, beyond which absorbance and flocculation continued to increase in the presence of Fe^2+^.

#### Interpretation

Lauric acid (LA) has a longer carbon chain (12 carbons) than capric acid (CA) (10 carbons). Although shorter carbon-chain-length fatty acids are known to reduce the stability of vesicles [46], we provide evidence for the opposite (Figure 9), i.e., that they increase the stability of vesicles (decrease flocculation). The temperature at which the analyses were conducted, and the different solubilities of lauric and capric acid, may explain this observation. Longer chain fatty acids require higher temperatures to dissolve, reflecting a phase transition in the properties of lipids. Beyond the temperature of lipid phase transition, lipids behave as a fluid rather than a gel. The phase transition temperature of capric acid is ~32 °C, and the phase transition temperature for lauric acid is ~44 °C [52]. The average ambient temperature recorded inside the anaerobic chamber during this study was 30.2 °C. Although samples were maintained at >45 °C in a water bath, sample cooling after removal from the bath may have resulted in some fatty acids becoming insoluble (Figure 9). The experimental temperature was sufficiently below the solubility limit/phase transition temperature for LA, such that the absorbance reading increased as insoluble solid white flocs appeared in solution. This implies that lipid vesicles made of shorter-chain length fatty acids, such as CA, may more readily form vesicles and resist de-solubilization at lower temperatures. The stability of different carbon-chain-length fatty acids is related to temperature and environmental conditions, and the fatty acid that forms the most stable vesicles is the one that has a carbon-chain length best suited to the environmental conditions.

### 3.5. Fatty Acids Combined in a ‘Mosaic’ Vesicle

**Figure 10:**
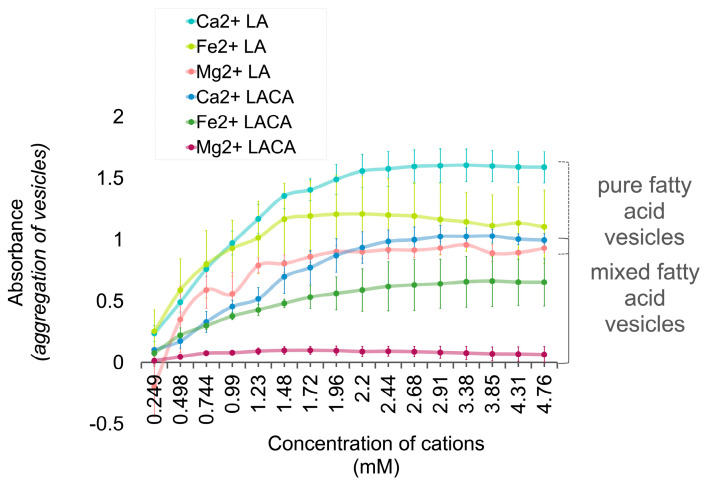
Flocculation of lipid vesicles with pure versus mixed ‘mosaic’ fatty acid compositions. Light absorbance of 10 mM LA and LACA lipid vesicle solutions with increasing cation concentrations, pH 7.5. High absorbance readings reflect high incidence of flocculation and unfavorable conditions for protocell formation. Samples were analyzed by spectrophotometry (400 nm) under anaerobic conditions. Uncertainty margins calculated as the standard deviation for *n* = 3 replicates.

Vesicles made of both LA and LACA had higher light-absorbance readings in the presence of Ca^2+^, Fe^2+^, and Mg^2+^ cations, with the relative effect of cations on flocculation being Ca^2+^ > Fe^2+^ > Mg^2+^. Vesicles of pure LA (lauric acid) had higher absorbance readings (i.e., more flocculation) than vesicles of LACA (lauric acid and capric acid) (Figure 10).

#### Interpretation

The inclusion of shorter-chain fatty acids in a membrane ‘mosaic’ reduced flocculation (i.e., stabilized vesicles) (Figure 10). The reduced incidence of flocculation observed in mosaic vesicles (LACA) relative to pure vesicles (LA) can be explained chemically by considering that mixed or ‘impure’ compounds do not form solids as readily as pure compounds do, due to melting-point depression [72]. Moreover, a fluid rather than a solid lipid phase is required to form membrane vesicles. When the composition of fatty acids in lipid vesicles is mixed, they are more soluble and stable, and behave as a fluid mosaic in a membrane structure. For example, vesicles made from an array of fatty acids, 1-alkanols, and isoprenoids have been shown to form stable vesicles even in seawater solutions [73], which typically severely disrupt vesicles [27]. The mixed-membrane feature is preserved in contemporary cells, where the cell membrane is composed not only of various lipids, but also of other compounds such as sterols and proteins [74,75].

### 3.6. Polymer and Lipid Co-Localization

**Figure 11:**
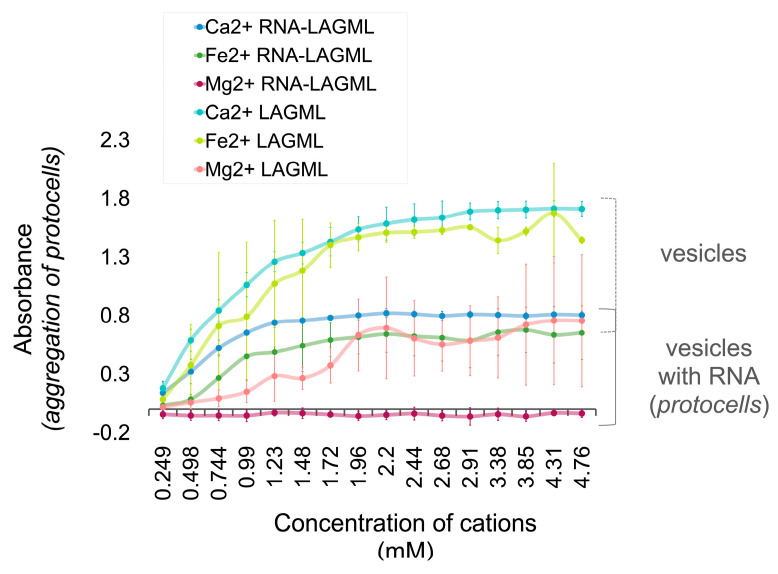
Lipid vesicle (LAGML) and protocell (RNA-LAGML) flocculation in the presence of cations. Light absorbance of LAGML and RNA-LAGML lipid vesicle solutions increase with increasing cation concentrations at pH 7.5. High absorbance readings reflect high incidence of flocculation and unfavorable conditions for protocell formation. Samples were analyzed by spectrophotometry (400 nm) under anaerobic conditions. LAGML was 10 mM in solution in a 4:1 ratio with RNA. Uncertainty margins are calculated as the standard deviation for *n* = 3 replicates.

**Figure 12:**
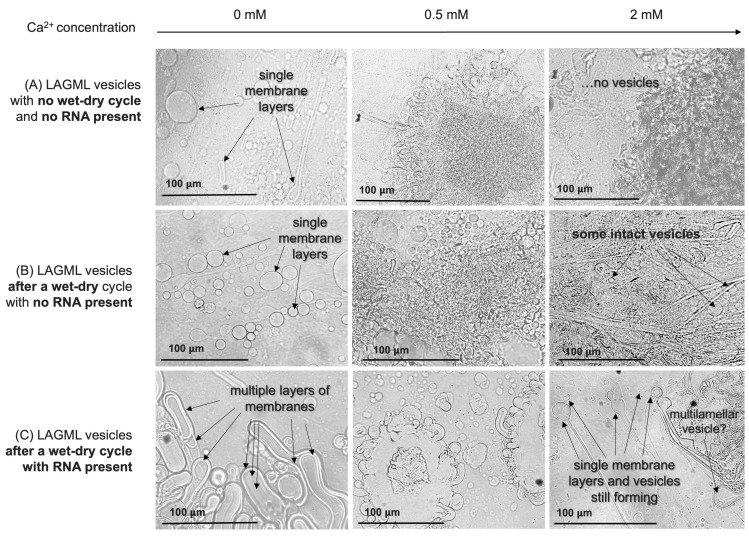
Light micrographs of flocculation of lipid vesicles caused by divalent cations, both with and without RNA present. (**A**): LAGML (100 mM) vesicles that did not undergo a wet–dry–wet cycle nor form in the presence of RNA. (**B**): LAGML (100 mM) vesicles that underwent a wet–dry–wet cycle without RNA present increasingly flocculate with greater Ca^2+^ exposure. (**C**): LAGML vesicles that underwent a wet–dry–wet cycle with RNA in solution appear to have multiple layers of membranes at low Ca^2+^ concentrations (0–0.5 mM), and reduced flocculation at high Ca^2+^ concentrations (2 mM) compared with vesicles in panel A. Note that RNA was not stained in this experiment, so its exact location is not confirmed by these micrographs.

LAGML vesicle samples (of lauric acid and glycerol monolaurate) that underwent one wet–dry–wet cycle in the presence of RNA (RNA-LAGML) had lower absorbance readings, i.e., less flocculation, than LAGML samples that did not contain RNA or undergo a wet–dry–wet cycle (Figure 11). RNA-LAGML samples still showed increased light-absorbance readings in the presence of high concentrations of Ca^2+^, Fe^2+^, and Mg^2+^ cations, with their relative effect on flocculation being Ca^2+^ > Fe^2+^ > Mg^2+^. The effect of divalent cations on vesicles that underwent a wet–dry–wet cycle in the absence of RNA can be seen in Figure 12B.

LAGML solutions which did not undergo a wet–dry–wet cycle and formed in the absence of RNA had observable vesicles only at 0–0.5 mM Ca^2+^ (Figure 12A). LAGML solutions which underwent a wet–dry–wet cycle, but did so in the absence of RNA, had observable vesicles from 0 to 2 mM (Figure 12B) amidst mostly flocs. LAGML solutions that underwent one wet–dry–wet cycle in the presence of RNA (RNA-LAGML) had observable vesicles from 0 to 2 mM Ca^2+^ under plain light microscopy (Figure 12C). RNA-LAGML vesicles in panel C show evidence for multiple layers of membranes in some cases (“multilamellar” membranes) at 0 mM, and intact membrane structures at 2 mM Ca^2+^.

#### 3.6.1. Interpretation

While it has been widely demonstrated that RNA can be encapsulated inside lipid vesicles to make protocells through wet–dry cycling [7,16,27,50,52], the exact location and full spectrum of the possible binding interactions of RNA and lipid membranes is not known, nor demonstrated in this study. Below, we describe three scenarios for RNA–lipid interactions that could explain Figure 11 and Figure 12.

##### RNA–Lipid Interactions during Wet–Dry Cycling

As lipid solutions are heated, water evaporates while the lipids and other dissolved components dry into a concentrated film. This evaporative process allows progressive molecular crowding to occur, while lipid vesicles in solution can fuse their membranes and create parallel layers of lipids; these can serve as ‘highways’ for molecular polymer transport in the intermediate phase between wet and dry [7]. Given that RNA monomers can bind to fatty acid vesicles made of capric acid [51] and concentrate inside vesicles [27,53], it is possible that the wet–dry cycling allows for RNA to come into close contact with the lipids, which allows binding to occur. This co-location and binding interaction could be preserved in the wet phase, whereby RNA bound to individual self-assembled lipid vesicles physically reinforces them, creating a greater stability for the vesicles with bound RNA in the face of cations in solution. This scenario is consistent with a similar observation reported in [51].

##### Morphological Changes/Multilamellar Structures

It is also possible that the binding interaction just described leads to the formation of multilamellar membranes (Figure 12C), which reinforce stability based on ‘contingency’ layers of membranes. Full polymer strands may interact with fatty acid membranes in a ‘gel phase’ between wet–dry cycles [7]—instead of individual RNA monomers binding to lipids as observed in [51]—which could keep multiple lipid membranes in close proximity upon rehydration, creating multilamellar structures (Figure 12C). In the presence of cations, the outermost membrane of the lipid vesicle alone could be subjected to cation binding, while the underlying membranes are protected and able to re-form membranes upon rehydration. The formation of multilamellar lipid vesicles thus introduces an advantage for protocells by providing additional layers of protection to internal contents, and thus could partially explain reduced flocculation, i.e., the increased stability of protocells (Figure 11 and Figure 12). The ‘outer layer’ feature of primitive cell membranes is conserved in modern cells, known as the ‘cell envelope’, in which multiple layers of lipids constitute an outer barrier to environmental changes [75,76].

##### Effect of Morphological Differences on Light Absorbance

Light absorbance and light scattering are sensitive not only to the degree of vesicle flocculation, but also to membrane morphology, size, and abundance of vesicles [77,78]. Lipid vesicles in the presence of RNA form larger vesicles with a larger internal volume of solution (Figure 12C). The resulting size and shape of the vesicles is referred to as ‘osmotic swelling’, due to high concentrations of nucleic acids inside vesicles [27,77,79]. Large vesicles have greater internal volumes and can allow more light to pass through, which could contribute to reduced turbidity and light absorbance observed via spectrophotometry in Figure 11. However, given that RNA–lipid vesicles may provide some resistance to flocculation through the formation of multilamellar structures, a combination of morphology and stability likely contribute to the reduced light absorbance recorded in this study. Additionally, multilamellar membranes can increase turbidity to a greater degree than small vesicle sizes do [78], which may contribute to an over-estimation of the flocculation assumed by turbidity in Figure 11, and an under-estimation of the stability provided by multilamellar vesicles in the face of cations.

### 3.7. Summary of Experimental Findings

**Figure 13:**
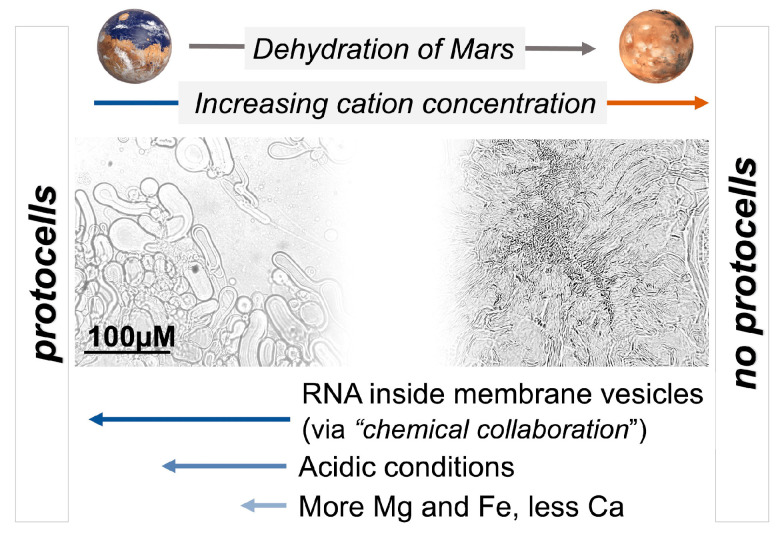
Schematic summary of the findings from this research. Arrows at the bottom indicate the relative mitigating impact of RNA, acidic pH, and specific cation compositions on protocell stability in the face of high concentrations of cations and reducing volumes of water.

All cations destabilize the fatty acid membranes tested in our study, but the relative extent of destabilization by different cations is determined by their unique ionic properties and interactions with water. During the dehydration of the surface of Mars ~3.5 Ga (Figure 2), the effect of cation concentration on flocculation would have increased. The resistance of lipid membranes to cations in solution is greatly affected by the physical and chemical conditions in the local environment and the properties of the individual lipids themselves. We expect resistance to flocculation to be variable between different types of lipids, but speculate that the relative impact of Ca^2+^ > Fe^2+^ > Mg^2+^ on flocculation would remain largely the same between lipids. Factors such as temperature, pH, and wet–dry cycling can create selective pressure driving the co-evolution of membranes and functional polymers, e.g., RNA, to form protocell communities on the path toward becoming living cells. This sequence of events could describe the origin of life on Earth, and potentially Mars. Novel experimental findings and a summary of results and discussion are schematically presented in Table 3 and Figure 13.

## 4. Discussion

Selection ‘hurdles’ for life on Earth were unique, as they would also have been on Mars and other Earth-like planets. It is important to consider the differences in selective pressures provided by these hurdles through time on different planets when considering the possibility of life arising on such planets.

### 4.1. Iron-Rich Geobiochemistry on Mars? Implications of the Relative Impact of Ca^2+^ Fe^2+^ and Mg^2+^ on Vesicle Stability

Biochemistry emerged from geochemistry [80], and if Mars had a high surface expression of iron, this could have shaped biochemistry on Mars as both a unique selection hurdle and a chemical evolutionary advantage for emerging protocell communities. Our findings indicate that iron has less of an effect on destabilizing vesicles than does calcium (Section 3.1). If a high relative abundance of iron over calcium is expected in the mineralogy of the Martian surface [81], Mars may have presented more favorable conditions for protocell formation than planets with more calcium-rich lithologies. Given that iron is thought to play an important role in RNA synthesis in primitive cellular life [47,48,49], we speculate that iron-based substrates may have acted as metal catalysts useful in the evolution of early polymers. This contention requires further investigation, but it does raise the question of when and how Fe^2+^ became important to the evolution of protocells if it did not interact with membranes to the same extent as its cationic counterparts.

Although Fe^2+^ did not destabilize vesicles in our experiments as much as Ca^2+^, Fe^2+^ still destabilizes lipid vesicles and forms salts that have an unknown impact on protocells. A balance between the destabilizing effects of iron on lipid vesicles and its function in catalyzing early prebiotic chemistry likely existed. While Fe^2+^ produced a flocculation of lipid vesicles, Fe^2+^ in the form of ferrous sulfate readily undergoes oxidation, even under the anaerobic conditions of our experiments, and produced precipitates (Section 2.1). This demonstrates the extreme redox sensitivity of iron, and suggests that in a natural setting it may readily form varied compounds and precipitates in a prebiotic environment, distinguishing its behavior from metal cations such as Ca^2+^ and Mg^2+^. Fe^2+^ precipitates also contribute to the turbidity of a solution, leading to more experimental variability in results and potentially an over-estimation of the amount of turbidity caused by flocculation. If a large proportion of Fe^2+^ free cations are binding with other compounds to produce salts, it is also possible that a lower concentration of total cations in natural settings was available to interact with anions, reducing the impact iron could have on the flocculation of lipid vesicles during a cellular origin-of-life process.

If the increased surface expression of iron on Mars translated to an increased incorporation or reliance on iron for biological chemistry, the oxidation of Mars at ~3.5 Ga would have been a major selection hurdle for life to overcome. Oxidation on Earth occurred much later, ~2.4–2 Ga [39], and was caused by a transition to photosynthetic metabolism in extant cyanobacteria. On Mars, by contrast, the reduced size and gravity of Mars made it more susceptible to atmosphere escape, resulting in greater UV radiation and photo-oxidation at the surface. Other factors such as meteorite impacts in the presence of water [82] also contributed to photo-oxidation processes. Regardless of the cause of the oxidation, if proto-cellular life was still in initial phases of evolution on Mars at 3.5 Ga, it may not have had a sufficient evolutionary foothold to adapt in the short timescale (3 to 3.5 Ga) that such changes in planetary redox conditions took place. Indeed, oxidizing planetary conditions would have severely impacted any biochemistry sensitive to redox states, e.g., most life on Earth depends on nitrogen being in a −3 oxidation state [30]). Although iron could provide both benefits and challenges in the evolution of protocells, the mass oxidation of the surface of Mars may have been a hurdle too large for early protocells with limited molecular technology to overcome.

### 4.2. The Dehydration of Mars: Implications from the Concentration of Cations in Natural Settings on Earth and Mars

The Martian hydrosphere was short-lived compared to that on Earth [30], and the total surface water volume on Mars diminished over time. In the context of this research, the loss of surface water on Mars implies that cation concentrations, presumably along with lipid concentrations, would have increased through time as the amount of water available to act as a solvent decreased (Figure 13). The adverse effects of cation–lipid binding, enhanced due to evaporative events creating higher cation concentrations, would have been a challenge for emerging protocells. Salt is to be included in this assessment of cations on Mars, as there is extensive evidence of evaporites and salty brine remnants on Mars, the formation of which is linked to the loss of episodic surface water, contributing to a scenario in which the concentration of salts and other cations at the surface continuously increased over time [40]. Salt has a dramatic osmotic effect on destabilizing membranes [27], and enhanced salinity as a consequence of evaporative conditions is another geochemical constraint that may have proved too great a selection hurdle for protocells on Mars to persist. These ionic challenges could be partially mitigated if life was emerging in volcanic hot springs rather than hydrothermal vents or impact-induced hydrothermal springs, because (a) precipitation cycles provide a source of distilled freshwater in pools where protocells might otherwise fail to persist, and (b) the hydrothermal plumbing in volcanic hot springs could have provided a refuge habitat [29] if cellular life was capable of such adaptation by ~3.5 Ga. The concentrations at which the effect of cation-induced flocculation becomes relevant are higher than observed in natural settings on Earth (Figure 6). Cation concentrations in natural settings could have provided a ‘healthy’ challenge for protocell survivability, driving the evolution of cation-tolerant protocells into cellular life, rather than being a source of major destruction. However, during a rapid loss of surface water on Mars, cation concentrations would have increasingly pushed protocells towards mass flocculation, such that cations could have posed a major selective barrier on Mars and not on Earth, which retained its surface water.

### 4.3. Selection of Cell Membranes on Earth-like Planets Such as Mars

#### 4.3.1. The Origin of Chemical and Biophysical Selection Processes

The origin of life is considered to occur through the pre-Darwinian evolution of chemical systems [7,16,17,18,19,20]. Before biology, the underlying processes of selection and evolution applied to individual molecular complexes at the chemical level. Conceptually, the evolution of a system can take place when there is (a) continuous variability in the components of the system, (b) an interaction of the components with the environment of the system, (c) the destruction or removal of some constituents from the system, while others persist and propagate, and (d) a mechanism that selects among the constituents and determines whether the constituent persists or perishes [83]. Selection is critical to the evolution of the system and need not be specific to the type of system it is acting upon. In prebiotic chemical systems, selection can also occur in the organic compounds that assembled before life began. Molecules are initially selected by the environment based upon their physical stability. Over time and with repeated environmental and chemical cycling, some chemical constituents are destroyed but not others, analogously to life and death cycles in biology (c). Certain molecules that persist through environmental and chemical cycles are better suited than others to persist in such chemical environments. The physical persistence of molecules provides the foundation for subsequent modifications and diversity (a), feedback loops (b), and the construction of more robust and complex structures, such as protocells. Biologically relevant molecules such as amino acids, nucleobases, and amphiphilic compounds can also be selected (d) based upon their potential function and behavior in a given chemical system.

#### 4.3.2. pH

pH conditions in natural hot-spring settings and between individual pools can vary greatly. For example, pH in the Hell’s Gate hot spring at Whangapipiro, New Zealand, ranges from pH 1.65 to 8.1 [52]. This variability encompasses the pH conditions conducive for lipid vesicle formation (pH near the pKa of fatty acids), flocculation, and metal-polymer binding (neutral-high pH), as well as flocculation evasion (sub-neutral-low pH). While low pH conditions may reduce flocculation risks for protocells, there is value in the diversity of pH conditions in the landscape, as without the challenging high pH conditions, there would be no driver for selection and evolution to flocculation resistance, e.g., polymer–lipid stabilizing interactions (Figure 10 and Figure 11). Selective barriers such as pH were essential to the evolutionary process of assembling protocells.

#### 4.3.3. Membrane Composition

**Figure 14:**
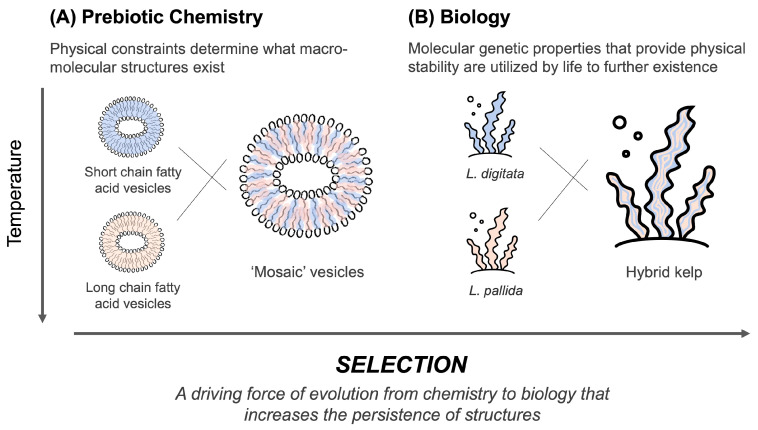
‘Hybrid vigor’, a concept from modern biology, echoes prebiotic chemistry. The characteristics of two individuals combined lets the hybrid take advantage of a wider range of genetic information when responding to a larger range of environmental conditions, from prebiotic chemistry (**A**), e.g., fatty acid mosaic vesicles, to modern biology (**B**), e.g., kelp [84].

‘Mosaic’ vesicles of a mixture of short-chain and long-chain fatty acids are more stable than pure vesicles in the presence of cations (Figure 10). This is attributed to the increased solubility of mixed chemical substrates, and that short chain fatty acids fared better in low temperatures than long chain fatty acids did, and vice versa. Scaling this relationship up to a conceptual parallel in modern biology, it helps to consider ‘hybrid vigor’. Hybrid vigor is a phenomenon in which hybridized individuals are more robust in certain environments than their individual predecessors, as they draw functionality from a more diverse genetic background. For example, the increased upper thermal tolerance in kelp hybrids arose from a crossing of *L. digitata* and *L. pallida* [84], low- and high-temperature-tolerant parents (Figure 14B). Prebiotic hybrids (panel A) in this research are mosaic fatty acid vesicles, in which a mixture of short (stable at lower temperatures)- and long (stable at higher temperatures)-chain fatty acids increase the stability of the vesicle over a broader range of temperatures. Hybrid vigor has conceptual parallels in biology and prebiotic chemistry, and its function was perhaps retained and amplified by biology through an evolutionary process and multi-scale selection.

### 4.4. Shift from Passive Selection to Functional Selection: Implications of RNA–Lipid Stabalising Interactions

The encapsulation of polymers inside lipid vesicles is a significant step in the origin-of-life transition, as selection processes shift from working on individual molecules alone toward an interacting set of molecules. The combination of RNA inside a vesicle creates an ‘emergent’ structure in chemistry, where their combined function as a larger ‘whole’ acted as a new subject of evolution. The stabilizing role RNA plays on the formation of lipid vesicles (Figure 11 and Figure 12; Section 3.6.1), speculated to be due to multilamellar membranes, is an important example of selection during the origin of cellular life. In this case, ‘passive selection’ based upon physical stability and molecular co-constraints provides transitions to ‘functional selection’ based upon the function of that interaction (which produces greater stability). This can also be described as a transition from ‘static persistence’ to ‘dynamic persistence’ by ‘first’- and ‘second’-order selection [85]. This study demonstrates the same theoretical shift experimentally in prebiotic chemistry. Free floating RNA is inherently unstable in an environment such as a hydrothermal system. Captured inside a protocell, however, it is protected from environmental stressors such as changes in pH and concentrations of ions. At the same time, RNA influences the morphology of the membrane, which contributes to the increased stability of membranous vesicles. The interaction is functional in enhancing persistence due to the mutually reinforcing chemical behavior of RNA and lipids, so it is ‘selected for’ by the environment. Their combination creates a robust macromolecular structure (a protocell) that provides a new stage for selection and evolution to operate on at the chemical level, along the pathway towards Darwinian selection.

Modern cells have ‘technologically’ advanced molecular machinery that increases cell-membrane stability by directing their synthesis, protection, regulation, and maintenance [76,86,87]. Although protocells lack such molecular machinery, it is possible that the genetic control of modern cell membranes is a highly evolved and expanded feature of the primitive effect found in this research, in which nucleic acids (and perhaps other polymers, peptides, and even RNA monomers alone [51]) prevented membrane collapse.

### 4.5. Searching for Life on Mars and Other Earth-like Planets: An Origin-of-Life-Informed Space-Exploration Strategy

Understanding the selection hurdles described above has critical implications for future exploration strategies that seek signs of ancient life on Mars. The most visited geologic sites of NASA rover missions to Mars are crater paleolakes, which were habitable but are not considered urable [1]. Terrestrial volcanic hot springs are considered urable, and a more promising geochemical environment for the origin of life on Earth [1,7,29,88,89]. Ancient hot-spring deposits have been identified on Mars that correspond to a period in the history of Earth when cellular life was thought to have originated [29,30]. However, if life could not emerge and form strong footholds on Mars between 4 and 3.5 Ga (Figure 2), it may not have had the molecular technology to adapt to and survive the rapidly changing planetary conditions that occurred after 3.5–3 Ga, let alone distribute from hot-spring settings and inhabit littoral and lacustrine regions such as the Jezero Crater [29]. If life did not reach the ‘distribution phase’ during the planetary origin-of-life transition [7], then potential origin-of-life sites such as Columbia Hills and other volcanic hot springs recognized in multiple locations on Mars would be the most strategic places to target for exploration [29,30,56,88,89]. Furthermore, the ability of surface hydrothermal environments to drive wet–dry cycles and facilitate polymer and peptide formation gives them an advantage over subsurface environments in forming cellular life [1]. An additional benefit to targeting these locations is that life on Mars would have had a strong evolutionary incentive to retreat underground through hydrothermal plumbing [29], and with high interest in searching for life in subterranean environments on Mars [90], these locations provide a promising pathway forward.

Searching for signs of ancient life on Mars continues to be a scientific priority [91]. Thus, it is imperative to more thoroughly investigate whether early Mars could have supported an origin of life, where this could have taken place, and if there was sufficient evolutionary opportunity to inhabit the surface. Otherwise, any search efforts are limited by the assumption that life originated on Mars, and rapidly expanded away from volcanic hydrothermal sites of origin to inhabit other aqueous environments such as crater lakes. Given the possibility that life did not have enough time or evolutionary incentive to extensively inhabit surface lakes, an understanding of origin-of-life processes in volcanic hydrothermal settings could guide mission strategies for future exploration missions to Mars.

The scientific community is continuing to search for signs of life on places habitable to life as we know it (e.g., Europa, Enceladus, and a growing number of exoplanets), as well as environments that could have hosted an origin of life similarly to Earth (e.g., early Mars). This study suggests narrowing the search for life to planetary bodies with complex, dynamic surface environments that can drive chemical selection, e.g., exposed volcanic hydrothermal pools. It is difficult to quantify the complexity and urability of planetary environments at present [1]. Nevertheless, we speculate that subsurface aqueous environments and hydrothermal vents on icy worlds, e.g., Europa and Enceladus, might suffer from reduced combinatorial complexity compared to the environments on the surfaces of Earth, Mars, Venus, and Titan, where geology interfaces with the atmosphere in addition to water and other liquids. We suggest it would be strategic to focus on searches for origin-of-life environments in and beyond the solar system that facilitate combinatorial chemical selection and complexity generation.

### 4.6. Future Work

This study highlights the noticeable differences in the effects of iron versus other cations on protocell stability and function, which suggests that divergence in evolutionary trajectories of cellular life under Martian conditions should be considered. Evident, too, are contrasts rather than similarities between early Mars and Earth in terms of how conducive each was to the origin of life. We suggest that future experiments investigate the effects UV, temperature, reduced gravity, and Fe^2+^ catalysts as additional Mars-specific variables to better characterize the complexity of the Martian environment. This contrasting approach would help enhance understanding of how life on Mars could differ from and be similar to life on Earth. Additionally, further characterization of the multilamellar membranes, perhaps employing cryogenic electron microscopy for this purpose, could be a fruitful avenue to pursue. Lastly, future investigations should determine the exact nature and location of the interaction between RNA and lipid membranes during vesicle formation, in order to understand the possible mechanisms of RNA-enhanced vesicle survivability. If RNA precursors provide lipid vesicles with physical resistance to environmental stressors (Section 3.6.1 and Section 4.4), this could be one of the earliest ‘functions’ of polymers in a protocell structure.

In strategies for future experimentation in this field, it is important to note that the experimental outcomes are completely dependent upon experimental conditions. Similarly, evolutionary outcomes are dependent upon environmental conditions. Many variables played a role in shaping the evolutionary trajectory of protocells, and those tested here were chosen to inform the effect of individual variables rather than simulate fully the complex origin-of-life environment. The reality of origin-of-life settings in natural systems provides a huge diversity of combinatorial conditions. Testing the effect of individual variables on individual molecules is inherently unrealistic, so the origin-of-life field as a whole would benefit from new scientific approaches able to characterize complex systems, such as ‘messy-chemistry’ approaches [92]. One aspect of messy chemistry is the generation of diversity. Diversity is the platform upon which selection can act, and selection fuels directional organization such as in living processes. As discussed in Section 4.4, selection can also act on the interaction between individual entities, and in a natural setting with innumerable interactions contributing to messy chemistry, how selection acts on a network of interactions is a characteristic of origin-of-life settings that cannot be ignored. Complex systems and messy-chemistry approaches could be key to conceiving and testing origin-of-life theories, because life is a process that organizes messy background chemistry through selective and evolutionary processes.

## 5. Conclusions

A fundamental connection exists between geochemical settings and the biological chemistry that originates from them. For planets to develop life from an early surface prebiotic chemistry phase, sufficiently dynamic and combinatorially complex environments capable of driving selection must be sustained. Hydrothermal systems are an example of settings that can accumulate and also mechanically combine organic molecules into a living system, through cycling and selection, e.g., cation exposure. The nature of the cyclic and selective environment of life on Earth’s origin is reflected and amplified by biology today, which is inherently cyclic and evolves through similar selection mechanisms thought to be present in hydrothermal systems during the origin of life [7].

This study highlights the dual nature of challenge and opportunity in the selection hurdles posed by hydrothermal settings, i.e., those which shape rather than prevent living outcomes. Instead of considering selection hurdles as challenges to avoid, and seeking habitable environments that would be pristine enough to cradle life as we know it, if we are to find life then we should seek environments that constantly challenge chemistry. It is the active ‘breaking’ that ‘makes’ life. Life cannot be described by prebiotic molecules or the environment they are in, but it can be described by the selective process that integrates those molecules into new structures and drives the evolution of chemistry itself to life.

## Figures and Tables

**Table 1:** Vesicle preparation reference key. Lipids were prepared for flocculation experimentation to make vesicles composed of mixtures of lauric acid (LA), capric acid (CA), glycerol monolaurate (GML), and RNA in buffer.

‘LAGML’ 10 mM	‘CAGML’ 10 mM
Lauric acid (LA, 12 carbons) and glycerol monolaurate (GML) (1:1 by weight) in 10 mM TEA pH 7.5	Capric acid (CA, 10 carbons) and glycerol monolaurate (GML) (1:1 by weight) in 10 mM TEA pH 7.5
‘LAGML (acidic)’ 10 mM	‘LA’ 10 mM
LAGML in 10 mM MES pH 5.5	Pure LA in 10 mM TEA pH 7.5
‘LACA’ 10 mM	‘RNA-LAGML’
LA and CA (1:1 by weight) in 10 mM TEA pH 7.5	LAGML and yeast RNA in a 4:1 ratio in 10 mM TEA pH 7.5, subjected to 1 wet–dry–wet cycle.

**Table 2:** Summary of the atomic properties that affect the interaction of Ca, Fe, and Mg with fatty acids and water.

	** 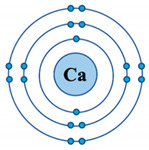 **	** 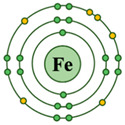 **	** 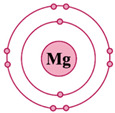 **
**Ionic Property**	**Calcium (Ca^2+^)**	**Iron (Fe^2+^)**	**Magnesium (Mg^2+^)**
D-orbital ( 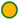 ) configuration	empty (0)	half (6)	empty (0)
Bond type	electrostatic	covalent	electrostatic
Ionic radius (Å)	0.99 [54], 1.12 [55]	0.74 [56], 0.78 [55], 1 [57]	0.65 [54], 0.76 [55]
Coordination number	6	6	6
Bond length of cation-O in H_2_O	2.46 [55]	2.12 [55], 1.98–2.05 [58]	2.10 [55]
Charge density	52 [59]	98–181 [59]	120 [59]

Ionic radius, bond length, and charge density values were sourced from [54,55,56,57,58,59].

**Table 3:** Experimental findings from this study in the context of previous investigations. Conditions for protocell formation and flocculation reported in this study are contextualized by previous studies [1,7,46,51,52], which formed the basis and impetus for this study.

Previous Work	This Work	
	Overall Trends	Novelty
Formation of protocells, sensitivity of fatty acid membranes to divalent cations, effect of pH on vesicle formation [1,7,46,51,52]; Flocculation or ‘flocculation’ of protocells in salt water [51].	Fe^2+^ destabilizes membrane vesicles less than does Ca^2+^, but more than Mg^2+^; Acidic pH reduces flocculation; Mixed fatty acid membranes resist flocculation.	Effect of Fe^2+^ on vesicle stability (with implications for Mars); Vesicles made of a mosaic of fatty acids are more stable in the presence of cations.
Individual nucleobases (monomers of RNA) bind to fatty acid membranes; Fatty acid membranes bound to nucleobases inhibit flocculation (flocculation in the presence of salt/Na^+^) [51].	Vesicles that underwent wet–dry cycling and encapsulated RNA resisted flocculation by divalent cations (Fe^2+^, Ca^2+^, and Mg^2+^).	RNA polymers stabilize fatty acid membranes; Wet–dry cycling in the presence of RNA produces large multilamellar vesicles.
Methods for assessing vesicle stability and flocculation: fluorescent microscopy, dye permeability, UV-Vis spectrophotometry.	Flocculation and stability quantified by measuring light absorbance with spectrophotometry.	Using spectrophotometry for evaluating the degree of flocculation exceeded expectations and proved a useful approach for quantitatively comparing impact of cations on flocculation.

## Data Availability

Data are contained within the article and [53].
